# Identification and validation of a multi‐assay algorithm for cross‐sectional HIV incidence estimation in populations with subtype C infection

**DOI:** 10.1002/jia2.25082

**Published:** 2018-02-28

**Authors:** Oliver Laeyendecker, Jacob Konikoff, Douglas E Morrison, Ronald Brookmeyer, Jing Wang, Connie Celum, Charles S Morrison, Quarraisha Abdool Karim, Audrey E Pettifor, Susan H Eshleman

**Affiliations:** ^1^ Laboratory of Immunoregulation NIAID, NIH Baltimore MD USA; ^2^ Division of Infectious Diseases Department of Medicine School of Medicine Johns Hopkins University Baltimore MD USA; ^3^ Department of Epidemiology Bloomberg School of Public Health Johns Hopkins University Baltimore MD USA; ^4^ Department of Biostatistics Bloomberg School of Public Health Johns Hopkins University Baltimore MD USA; ^5^ Department of Biostatistics UCLA School of Public Health Los Angeles CA USA; ^6^ Vaccine and Infectious Disease Division SCHARP‐FHCRC Seattle WA USA; ^7^ Departments of Global Health, Medicine, and Epidemiology University of Washington Seattle WA USA; ^8^ Clinical and Epidemiologic Sciences Durham NC USA; ^9^ Centre for the AIDS Programme of Research in South Africa (CAPRISA) University of KwaZulu‐Natal Durban South Africa; ^10^ Department of Epidemiology Mailman School of Public Health Columbia University New York NY USA; ^11^ Department of Epidemiology University of North Carolina Chapel Hill NC USA; ^12^ Carolina Population Center University of North Carolina Chapel Hill NC USA; ^13^ Medical Research Council/Wits Rural Public Health and Health Transitions Research Unit School of Public Health Faculty of Health Sciences University of the Witwatersrand Johannesburg South Africa; ^14^ Wits Reproductive Health and HIV Institute University of the Witwatersrand Johannesburg South Africa; ^15^ Department of Pathology School of Medicine Johns Hopkins University Baltimore MD USA

**Keywords:** Cross‐sectional incidence testing, Southern Africa, Subtype C, Women, Epidemiology

## Abstract

**Introduction:**

Cross‐sectional methods can be used to estimate HIV incidence for surveillance and prevention studies. We evaluated assays and multi‐assay algorithms (MAAs) for incidence estimation in subtype C settings.

**Methods:**

We analysed samples from individuals with subtype C infection with known duration of infection (2442 samples from 278 adults; 0.1 to 9.9 years after seroconversion). MAAs included 1‐4 of the following assays: Limiting Antigen Avidity assay (LAg‐Avidity), BioRad‐Avidity assay, CD4 cell count and viral load (VL). We evaluated 23,400 MAAs with different assays and assay cutoffs. We identified the MAA with the largest mean window period, where the upper 95% confidence interval (CI) of the shadow was <1 year. This MAA was compared to the LAg‐Avidity and BioRad‐Avidity assays alone, a widely used LAg algorithm (LAg‐Avidity <1.5 OD‐n + VL >1000 copies/mL), and two MAAs previously optimized for subtype B settings. We compared these cross‐sectional incidence estimates to observed incidence in an independent longitudinal cohort.

**Results:**

The optimal MAA was LAg‐Avidity <2.8 OD‐n  +  BioRad‐Avidity <95% + VL >400 copies/mL. This MAA had a mean window period of 248 days (95% CI: 218, 284), a shadow of 306 days (95% CI: 255, 359), and provided the most accurate and precise incidence estimate for the independent cohort. The widely used LAg algorithm had a shorter mean window period (142 days, 95% CI: 118, 167), a longer shadow (410 days, 95% CI; 318, 491), and a less accurate and precise incidence estimate for the independent cohort.

**Conclusions:**

An optimal MAA was identified for cross‐sectional HIV incidence in subtype C settings. The performance of this MAA is superior to a testing algorithm currently used for global HIV surveillance.

## 1 Introduction

Accurate methods for estimating HIV incidence are critical for HIV surveillance and for evaluating the effectiveness of HIV prevention efforts 1. Traditional longitudinal cohort studies are costly and may not include or retain individuals at high risk for HIV acquisition 2, 3, 4. This is particularly true in southern Africa, a subtype C endemic area 5 with high rates of population migration 6.

Cross‐sectional HIV incidence estimation offers an alternative to traditional cohort studies. Cross‐sectional incidence estimation uses biomarkers to identify individuals who are likely to have recent HIV infection 4. Most methods include serologic assays that measure the antibody response to HIV infection 4. Use of a single assay approach to estimate HIV incidence has been problematic, since viral suppression and AIDS can cause these tests to have values that are associated with recent infection 7. In contrast, multi‐assay algorithms (MAAs) that combine serologic and non‐serologic assays, such as viral load (VL) and CD4 cell count, have been identified that provide accurate incidence estimates in subtype B settings 8, 9, 10. These MAAs have been used to estimate HIV incidence in clinical trials and cohort studies 10, 11, 12, 13.

Many MAAs used in previous studies included the BED capture enzyme immunoassay (EIA) 14, which is being phased out. A limiting antigen avidity assay (HIV‐1 LAg‐Avidity EIA, SEDIA Biosciences Corporation Portland, OR and Maxim Biomedical, Bethesda, MD) 15, is now widely used for HIV incidence estimation in surveillance and research studies. The manufacturers of the LAg‐Avidity assay recommend using the assay in an algorithm where individuals with VL <1000 copies/mL are classified as having non‐recent infection 16. This algorithm is widely used to estimate HIV incidence in cross‐sectional surveys 17, 18, 19, and is currently being used in large surveys conducted as part of the PEPFAR‐supported Population‐based HIV Impact Assessment (PHIA, http://icap.columbia.edu/global-initatives/the-phia-project/). Many countries included in these surveys are in subtype C endemic areas 5. HIV incidence estimates obtained using this approach were recently presented for Zimbabwe, Malawi and Zambia (Conference on Retroviruses and Opportunistic Infections; http://www.croiwebcasts.org/p/2017croi/croi33590).

In this report, we evaluated the performance of assays and MAAs for HIV incidence estimation in subtype C settings. An optimal subtype C MAA was identified using pre‐determined performance criteria. We compared the performance of this MAA to five other testing algorithms (the LAg‐Avidity assay alone, the BioRad‐Avidity assay alone, a widely‐used LAg algorithm, and two MAAs previously optimized for subtype B settings).

## 2 Methods

### 2.1 Ethics statement

Written informed consent was obtained from study participants and studies were reviewed and approved by relevant institutional review boards. This study used stored samples from individuals who consented to future use of their specimens for research. No new samples were collected for this work. The institutional review board of the Johns Hopkins University approved the study of cross‐sectional incidence testing using stored study samples. The research was conducted in accordance with the principles expressed by the Declaration of Helsinki.

### 2.2 Samples used for analysis

The sample set used to identify an optimal MAA included 2442 samples from 278 participants with known duration of infection (0.1 to 9.9 years after seroconversion, Table 1). The samples were obtained from three studies that evaluated interventions for HIV prevention. The first study evaluated a vaginal microbicide in KwaZulu Natal, South Africa (CAPRISA) 20. The second study evaluated hormonal contraception and HIV infection in Zimbabwe and Uganda (Family Health International [FHI] 360); all but three of the samples from this study were from Zimbabwe. The third study evaluated herpes simplex virus type 2 treatment in Zambia (HIV Prevention Trials Network [HPTN] 039) 21. Participants in the three studies were HIV‐uninfected at enrollment and were tested for HIV infection at intervals ≤6 months; HIV RNA testing was performed retrospectively at the visit prior to HIV seroconversion to determine if participants had acute HIV infection at that visit.

**Table 1:** Study cohorts

Characteristic	Cohort		
	CAPRISA	FHI‐360	HPTN 039
Country of origin	South Africa	Zimbabwe^a^	Zambia
Number of samples	518	1839	85
Number of unique subjects	90	162	25
Range of duration of infection in years	0.06 to 3.7	0.04 to 9.9	0.15 to 0.8
Mean samples per subject (range)	6 (1 to 7)	12 (1 to 20)	4 (1 to 4)
Female sex, % of subjects	100%	100%	100%
Number samples from subjects on ART (%)	12 (2.2%)	220 (11.3%)	0 (0%)
Duration of infection in years			
0.0 to 0.5	159	306	42
0.5 to 1.0	173	262	43
1.0 to 2.0	88	448	0
2.0 to 3.0	76	105	0
3.0 to 5.0	22	347	0
≥ 5.0	0	371	0
CD4 cell count			
>500	228	685	54
500 to 200	271	822	26
<200	14	104	0
missing	5	228	5
Viral load (copies/mL)			
>10,000	260	560	37
10,000 to 1000	161	278	26
<1000	92	227	19
missing	5	774	3

a All participants from South Africa, Zimbabwe and Zambia were assumed to have subtype C infection based on the prevalence of subtype C in those countries. The FHI‐360 cohort included one individual from Uganda with three samples. That individual was infected with HIV subtype C based on subtype assessment of the *pol* region.

An additional sample set was obtained from an independent, longitudinal cohort study that evaluated the impact of conditional cash transfer on HIV acquisition in young women in South Africa (HPTN 068) 22. The study was conducted from 2012 to 2015. Samples collected in 2014 were used for cross‐sectional incidence estimation; results were compared to the observed longitudinal incidence in the cohort. This analysis included 1360 participants (1269 HIV‐uninfected and 91 HIV‐infected participants; 61 participants were infected in 2013 or earlier).

### 2.3 Laboratory methods

The LAg‐Avidity assay was performed according to manufacturer's instructions 15 with one modification. Samples with values <2.0 normalized optical density units (OD‐n) were tested in duplicate. The Johns Hopkins modified BioRad‐Avidity assay is based on the Genetic Systems 1/2 + O ELISA (Bio‐Rad Laboratories, Redmond, WA); testing was performed with this assay as previously described 23. Briefly, samples were diluted 1:10 and tested in duplicate. Diluted samples were incubated for 30 minutes at 37°C with or without the chaotropic agent, diethylamine (DEA; diluted in deionized water). The avidity index (AI) is calculated as the result for the DEA‐treated well divided by the result for the non‐treated well, times 100. LAg‐Avidity and BioRad‐Avidity testing was performed at Johns Hopkins University in Baltimore, Maryland. HIV viral load and CD4 cell count data were obtained from the primary studies.

### 2.4 Statistical methods

The statistical methods used in this report are outlined below, and are described in more detail in previous publications 8, 9, 24. A S3 provides further information, including a description of statistical adjustments that were used to account for missing data.

#### 2.4.1 *Identification of assay‐positive and MAA‐positive samples*

Assays and MAAs were used to identify individuals who were likely to have been infected near the time of sample collection (assay‐ or MAA‐positive). For individual assays (LAg‐Avidity assay, BioRad‐Avidity assay), samples were classified as assay‐positive if the test result was above (VL and CD4) or below (LAg‐Avidity and BioRad‐Avidity) cutoffs.

A range of possible cutoffs were evaluated. Twenty‐six cutoffs were evaluated for the LAg‐Avidity assay: 0.5 OD‐n to 3 OD‐n in increments of 0.1 OD‐n. Eight cutoffs were evaluated for the BioRad‐Avidity assay: 30%, 35%, 40%, 80%, 85%, 90%, 95% and 100% Avidity Index (AI); BioRad‐Avidity cutoffs between 40% and 80% were not evaluated, since the assay has poor reproducibility in this range. Eight cutoffs were evaluated for CD4 cell count: 50, 100, 150, 200, 250, 300, 400 and 500 cells/mm^3^. Nine cutoffs were evaluated for viral load: 400, 600, 800, 1000, 1500, 2000, 3000, 5000 and 10,000 copies/mL. The cutoff of 400 copies/mL HIV RNA was chosen because that was the lower limit of detection for the viral load assay used for analysis. A viral load cutoff of 1000 copies/mL is another important variable to consider when evaluating MAAs, since it is the lower limit of detection for viral load testing from dried blood spots (DBS). For MAAs, all possible combinations of these cutoffs were evaluated.

#### 2.4.2 *Performance characteristics of assays and MAAs*

The performance characteristics of individual assays (LAg‐Avidity alone; BioRad‐Avidity alone) and MAAs were evaluated by estimating the proportion of samples classified as assay‐ or MAA‐positive, as a function of time after seroconversion, [denoted *ϕ(t)*]. The values of *ϕ(t)* depend on the assay/MAA being evaluated. Estimating this function required first imputing each participant's seroconversion date, which is only known to be sometime in the interval between the last HIV‐negative visit and first seropositive visit (the “seroconversion window”). If HIV RNA testing indicated that the participant was acutely infected at the last HIV‐negative visit, the seroconversion date was estimated as 28 days after that visit. For all participants, infection times were sampled from a uniform distribution over the seroconversion window. Logistic regression models with cubic polynomials were then used to estimate *ϕ(t)* for each assay or MAA. The seroconversion dates were imputed 1000 times per subject, and the coefficients of the resulting *ϕ(t)* curves were averaged.

The estimated *ϕ(t)* function was then used to calculate the mean window period (i.e. the average duration of time an individual was MAA positive) and the shadow, which measures how far back in time incidence is measured 25. Confidence intervals (CIs) for these two characteristics were obtained using a bootstrap procedure, which was stratified by cohort and clustered by individual to account for correlations between samples drawn from the same person over time 8. An assay or MAA was considered suitable for HIV incidence estimation only if the predicted proportion of individuals classified as assay‐ or MAA‐positive 9.5 years after seroconversion was <0.001. An optimal MAA was selected using the following criteria: highest estimated mean window period among the MAAs that had an upper 95% CI for the shadow of <365 days. CIs for differences between the mean window period and shadow in MAAs were also constructed by bootstrapping.

The statistical accuracy of an algorithm for determining current incidence can be assessed by the variance and bias of the incidence calculated from the algorithm 25. We have previously shown that the variability is minimized by a criterion that selects the algorithm with the largest mean window period. However, that criterion must be balanced by the desire to estimate current incidence rather than incidence in the distant past. We have shown that the shadow can be used to determine how far back in the past incidence is measured 7, 9, 25. To estimate incidence within 1 year of the survey, we required that the upper 95% CI for the shadow of each algorithm was less than 1 year. Among algorithms that met this requirement, we selected the algorithm that had the largest mean window period 7, 9, 25.

**Figure 1:**
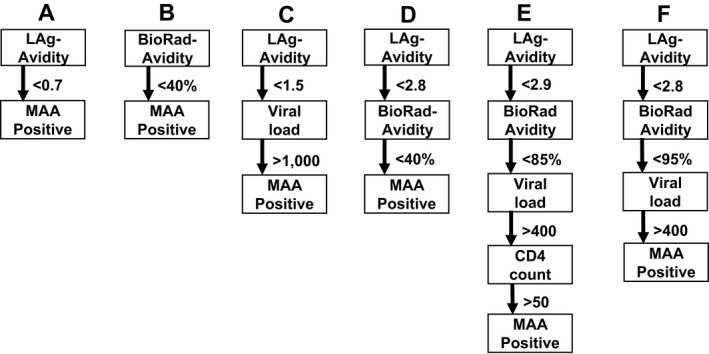
Assays and multi‐assay algorithms for cross‐sectional HIV incidence estimation. The figure shows the assays and cutoff used for six different testing methods: two individual assays, the LAg‐Avidity assay **(A)** and the BioRad‐Avidity assay **(B)**, the current testing algorithm recommended for the LAg‐Avidity assay **(C)**, two MAAs previously optimized for incidence estimation in subtype B settings **(D and E)**, and the optimal subtype C MAA identified in this report **(F)**. The units used for the assays were: LAg‐Avidity assay: normalized optical density units (OD‐n): BioRad‐Avidity assay: avidity index (%); viral load: HIV RNA copies/mL; CD4 cell count: cells/mm^3^. Individuals with the following results were classified assay‐ or MAA‐positive: **(A)** LAg‐Avidity <0.7 OD‐n; **(B)** BioRad‐Avidity <40%; **(C)** LAg‐Avidity <1.5 +  viral load (VL) >1,000; **(D)** LAg‐Avidity <2.8 OD‐n  +  BioRad‐Avidity <40%; **(E)** LAg‐Avidity <2.9 OD‐n  +  BioRad‐Avidity <85% + VL >400 +  CD4 > 50; **(F)** LAg‐Avidity <2.8 OD‐n  +  BioRad‐Avidity <95% + VL >400.

Individual assays and MAAs were further evaluated by estimating HIV incidence using cross‐sectional data from an independent cohort study (HPTN 068). This sample set was only used to compare cross‐sectional incidence estimates obtained with MAAs to the incidence observed in this longitudinal cohort. In this analysis, the cross‐sectional incidence estimator was (in % per year)$\begin{array}{c} I_{\mathrm{CS}}=\text{number of samples classified as assay or MAA positive}/\left(\text{number of HIV-negative samples}\right)\\ \times (\text{mean window period in years})\times 100\% \end{array}$ 7, 26. The CIs calculated for these estimates accounted for uncertainty in the mean window periods 3. The percent error of the cross‐sectional incidence estimates was determined by comparing these results to the observed longitudinal incidence estimate in the HPTN 068 cohort (*I*_cohort_), using the formula $\text{Error}=\frac{\left|I_{\mathrm{cs}}-I_{\mathrm{cohort}}\right|}{I_{\mathrm{cohort}}}\times 100\%$. The two avidity assays and four MAAs were included in this evaluation (Figure 1).

## 3 Results

A set of 2442 subtype C samples from individuals with known duration of infection was used to evaluate assays and MAAs for cross‐sectional HIV incidence estimation in subtype C settings. A total of 23,400 different algorithms were evaluated, 687 of these MAAs did not converge to zero during the period evaluated. Of the 22,713 MAAs that did converge, 3213 were excluded because their shadow was >1 year. The window periods and shadows for different testing algorithms evaluated are presented in the S1. Additional information for selected algorithms is presented in the S2. Among the remaining 19,500 algorithms, the one with the largest window period and highest single serologic cutoffs were further evaluated. For individual assays, the highest cutoff values that yielded shadows with an upper 95% confidence limit of <1 year were 0.7 OD‐n for the LAg‐Avidity assay and 40% AI for the BioRad‐Avidity assay.

**Figure 2:**
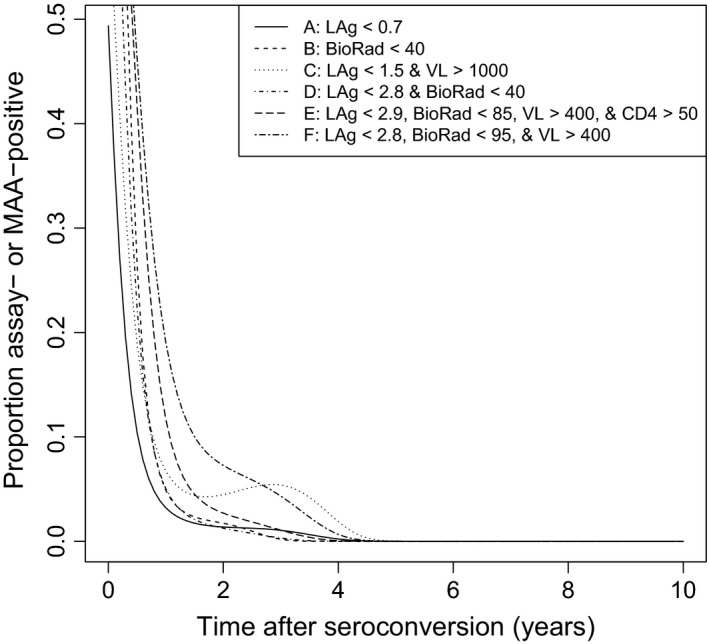
Modeled probabilities of an individual being classified as assay‐ or MAA‐positive as a function of duration of infection. The figure shows modeled probability curves of samples being classified as assay‐positive using the LAg‐Avidity assay or BioRad‐Avidity alone, or multi‐assay algorithm (MAA)‐positive using one of four MAAs.

Six testing algorithms were compared (Figure 1): (A) the LAg‐Avidity assay alone with an optimized assay cutoff (>0.7 OD‐n); (B) the BioRad‐Avidity assay alone with an optimized assay cutoff (<40% AI); (C) a widely used LAg algorithm; (D) a 2‐assay MAA previously optimized for incidence estimation in subtype B settings 24; (E) a 4‐assay MAA previously optimized for incidence estimation in subtype B settings 24; and (F) the optimal subtype C MAA identified in this report. The optimal subtype C MAA was selected from among 23,400 MAAs that included 1 to 4 assays with different assay cutoffs. The optimal MAA, based on the longest window period and a shadow <1 year, included the LAg‐Avidity assay, the BioRad‐Avidity assay, and viral load (Figure 1F). Figure 2 shows the proportion of samples classified as assay‐ or MAA‐positive as a function of time after seroconversion for each testing algorithms; the proportion converged to zero by 5 years for all six algorithms.

The performance characteristics of the six testing algorithms are summarized in Table 2. The optimal subtype C MAA had the highest mean window period: 248 days (95% CI: 215, 282); this was significantly higher than the mean window period for a LAg‐viral load algorithm (algorithm C): 142 days (95% CI: 118, 167). The shortest mean window period was obtained for the LAg‐Avidity assay alone: 71 days (95% CI: 57, 86). The shadows for five of the six testing algorithms were <1 year, and the CIs for many of the algorithms overlapped. The shadow for the current LAg algorithm (C) was 410 days (95% CI: 318, 491), which was greater than a year. The optimal MAA (F) and the current LAg algorithm (C) had overlapping CIs for their shadows; however, the window period was significantly larger for the optimal MAA.

**Table 2:** Performance of HIV incidence testing algorithms

Algorithm		Window period^a^	Shadow^a^	HPTN 068 Estimate^b^	Error^c^
A	LAg <0.7	71 (57, 86)	237 (162, 324)	3.7 (1.6, 7.2)	92%
B	BioRad <40	151 (135, 169)	146 (112, 181)	2.5 (1.3, 4.3)	30%
C	LAg <1.5 + VL >1,000	142 (118, 167)	410 (318, 491)	2.4 (1.2, 4.4)	28%
D	LAg <2.8 + BioRad <40	126 (108, 144)	152 (114, 193)	3.0 (1.6, 5.2)	56%
E	LAg <2.9 + BioRad <85 + VL >400 + CD4 > 50	191 (168, 217)	201 (159, 245)	2.6 (1.5, 4.2)	35%
F	LAg <2.8 + BioRad <95 + VL >400	248 (215, 282)	306 (256, 356)	2.1 (1.2, 3.4)	10%

a The window period and shadow are shown for each testing algorithm (A‐F); these variables are presented in days with 95% confidence intervals (CI) in parentheses. Units for assay cutoffs are: LAg‐Avidity assay: normalized optical density units (OD‐n); BioRad‐Avidity assay: avidity index (%); viral load: HIV RNA copies/mL; CD4 cell count: cells/mm^3^.

b Cross‐sectional estimates of annual HIV incidence in HPTN 068 in the 2014 survey year are shown for each testing algorithm; 95% CI are shown in parentheses. The observed longitudinal incidence in HPTN 068 in the 2014 survey was 1.9% (95% CI: 1.3, 2.7).

c The error of the cross‐sectional HIV incidence estimate (compared to observed longitudinal incidence) is shown for each testing algorithm.

LAg: LAg‐Avidity assay; BioRad: BioRad‐Avidity assay; VL: viral load; CD4: CD4 cell count.

Each of the six testing algorithms was also used to estimate HIV incidence in an independent cohort, HPTN 068. During the 2014 survey in this study, the observed incidence based on longitudinal follow‐up (seroconversion) was 1.9% per year (95% CI: 1.3, 2.7). The number of individuals classified as recently‐infected for each algorithm was: A: 9, B: 13, C: 12, D: 13, E: 17 and F: 18. The point estimates of incidence obtained using the six testing algorithms ranged from 2.1% to 3.7% (Table 2); these estimates were all higher than the observed longitudinal incidence estimate. The optimal subtype C MAA had the most accurate incidence estimate (2.1%, 95% CI: 1.2, 3.5), which was within 10% of the observed longitudinal incidence. This MAA also provided the most precise incidence estimate, with the smallest CIs.

## 4 Discussion

We used a large set of subtype C samples from individuals with known duration of infection to identify an optimal MAA for HIV incidence estimation in subtype C settings. The optimal subtype C MAA, which included the LAg‐Avidity assay, the BioRad‐Avidity assay, and viral load, had a mean window period of 248 days. This is >100 days longer than the mean window period for a LAg algorithm (LAg‐Avidity assay <1.5 OD‐n  +  viral load >1000), which is widely used for incidence estimation in surveillance and other studies. Longer window periods for incidence assays identify a greater number of recently infected individuals and provide more accurate incidence estimates. In our study, Algorithm A identified nine recently‐infected subjects in the HPTN 068 confirmation cohort, while Algorithm F identified eighteen. Algorithms with larger window periods would allow for greater precision of incidence in national surveys, the potential for regional analysis within these surveys, and the analysis of factors associated with recent infection. The optimal subtype C MAA had a shadow <1 year, indicating that it estimates incidence for a period within a year of sample collection. This optimal clade C algorithm provided the most accurate and most precise estimate of incidence in an independent, longitudinal cohort. Because the optimal MAA does not include CD4 cell count, it can be used to estimate incidence using stored plasma or serum samples. The PEPFAR PHIA surveys collect data for the LAg‐Avidity assay and viral load; the optimal subtype C MAA identified in this report could be used to refine incidence estimates from those studies by testing a subset of the stored samples with the BioRad‐Avidity assay (i.e. those with LAg‐Avidity <2.8 and viral load >400). The mean window period for the optimal subtype C MAA is 1.75 times longer than the window period for the current LAg algorithm. Therefore, this MAA would identify more individuals with recent infection and would provide more precise incidence estimates. For these reasons, it may be possible to use the optimal subtype C MAA to obtain regional incidence assessments in the PHIA surveys or to compare incidence among various groups within the survey populations. The longer mean window period of the optimal subtype C MAA would also allow for smaller sample sizes in clinical trials evaluating interventions for HIV prevention 27.

Other studies have used the mean duration of recent infection (MDRI) to assess performance of methods for cross‐sectional incidence estimation. The MDRI and mean window period are determined by the area under the probability curve. Both determine the average time that individuals appear to be recently infected. The values for the MDRI and mean window period differ since the MDRI curve is typically truncated at two years after seroconversion; in contrast the mean window period is calculated as the area under the entire curve. The mean window period we calculated for the current LAg algorithm in this study (142 days) was nearly identical to the 2 year MDRI for this algorithm that was calculated by the Consortium for the Evaluation of the Performance of HIV Incidence Assays (CEPHIA; 141 days, 95% CI: 123 to 160 days) 28; the CEPHIA estimate was based on a sample set that was not limited to subtype C samples. A recent report cited a much lower MDRI for the LAg‐Avidity assay (101 days) 29.

In this report, we also used the subtype C sample set to analyse the performance of two MAAs that were previously optimized for incidence estimation in subtype B settings in low, medium and high incidence settings with different study populations 9. The optimal subtype B MAA provided a mean window period for the subtype C sample set that was longer than the mean window period for the widely‐used LAg algorithm and had shadows <1 year. In contrast, the current LAg algorithm had a shadow of 410 days, indicating that it estimates incidence for a period more than a year before sample collection. The annual incidence estimate obtained for the independent cohort study using the current LAg algorithm was more accurate and more precise than the estimates obtained using subtype B MAAs. However, it was less accurate and less precise than the incidence estimate obtained using the optimal subtype C MAA.

One limitation of this study is that all of the participants included in this report were women. Furthermore studies are needed to evaluate MAAs in subtype C cohorts that include men. The incidence in the HPTN 068 cohort was also fairly high (1.9% per year) and the population had a relatively low HIV prevalence (6.7%). In addition, the confidence intervals of all six algorithms overlapped, which was not surprising as the results were not statistically independent. Simulation studies could be used to validate the optimal MAA identified in this report under conditions that vary HIV incidence 11. Theoretically, algorithms with shadows >1 year may not perform well in settings with decreasing and increasing incidence, since they would over‐ and under‐estimate incidence, respectively 30. Confirmation cohorts with such conditions should be evaluated to further validate cross‐sectional incidence algorithms.

Furthermore research is also needed to identify MAAs for use in settings with other prevalent HIV subtypes, with circulating recombinant forms, and with mixed HIV strains (e.g. in Asia, where HIV‐1 B, CRF01_AE and many B‐C recombinant forms are observed 31). Development and validation of MAAs for use in East African settings may be particularly difficult, since individuals with subtype D infection have been shown to have delayed antibody maturation compared to other subtypes 32, 33. We previously demonstrated that HIV diversity is a useful biomarker for cross‐sectional incidence estimation 34. MAAs that include a combination of serologic measures, diversity measures, and other non‐serologic biomarkers (e.g. viral load) may be needed to obtain accurate incidence estimates in settings that include a high proportion of subtype D infections. Furthermore, validation of MAAs using DBS samples is critical, since DBS samples are easier to collect and prepare than blood samples obtained by phlebotomy. An initial study demonstrated that similar results were obtained for serological incidence assays using plasma and DBS samples stored at −80°C 35. Additional studies are needed to determine if the storage conditions of DBS samples impacts the performance of serologic incidence assays.

In summary, we used a large sample set from individuals with known duration of infection to identify an optimal MAA for cross‐sectional incidence estimation in subtype C settings. This MAA has a mean window period of 248 days and provided accurate and precise incidence estimates for an independent cohort. Because this MAA can be performed using stored specimens without CD4 cell count data, it could be used to refine incidence estimates from large population‐level surveys, such as those from the PEPFAR PHIA programme, with minimal additional testing. Furthermore studies are underway to evaluate this MAA in diverse study populations with varied HIV incidence.

## Competing interests

The authors have no competing interests to report.

## Author contributions

All of the authors contributed to preparation of the manuscript. Additional author roles are shown in the table below.

**Table:** 

Oliver Laeyendecker	Conceived of the study, responsible for sample testing, drafted the manuscript
Jacob Konikoff	Performed statistical analyses
Douglas E. Morrison	Performed statistical analyses
Ronald Brookmeyer	Lead statistician for this project
Jing Wang	Statistician for HPTN 039 and HPTN 068
Connie Celum	Principal investigator for the HPTN 039 study
Charles S. Morrison	Principal investigator for the FHI360 study
Quarraisha Abdool Karim	Principal investigator for the CAPRISA study
Audrey E. Pettifor	Principal investigator for the HPTN 068 study
Susan H. Eshleman	Conceived of the study, drafted the manuscript

## Disclaimers

The findings and conclusions in this article are those of the authors and do not necessarily represent the views of the National Institutes of Health. Use of trade names is for identification purposes only and does not constitute endorsement by the National Institutes of Health and Prevention or the Department of Health and Human Services. The views expressed here do not necessarily reflect the official policies of the City and County of San Francisco, nor does mention of the San Francisco Dept. of Public Health imply its endorsement.

## Funding

This study was funded by (1) the National Institute of Allergy and Infectious Diseases (NIAID), National Institutes of Child Health and Human Development (NICH/HD), National Institute of Drug Abuse (NIDA) and the National Institute of Mental Health (NIMH), Office of AIDS Research, National Institutes of Health (NIH), Department of Health and Human Services (UM1‐AI068613), and 2 R01‐AI095068 (NIH, NIAID). Additional support was provided by the Division of Intramural Research, NIAID, NIH.

## Previous publication or presentation of data

The data was presented at the Conference on Retroviruses and Opportunistic Infections, Seattle, WA. 2017, poster #879.

## Supporting information


**Figure S1.** THE figures shows the shadow and mean window period for 23,997 ALgorithms evalated.

**Table S1.** The table shows the assay cutoffs and performance characteristics of testing algorithms A through F, and 26 additional testing algorithms. VL: viral load (copies/mL); CD4: CD4 cell count (cells/mm^3^); values for the window period and shadow are shown in days

**Appendix S1.** Supplementary material.
