# Supplementary figures and images for: Identification and validation of a multi‐assay algorithm for cross‐sectional HIV incidence estimation in populations with subtype C infection
Source: J Int AIDS Soc. 2018 Feb 28;21(2):e25082. doi: 10.1002/jia2.25082 (PMC5829581; doi:10.1002/jia2.25082)

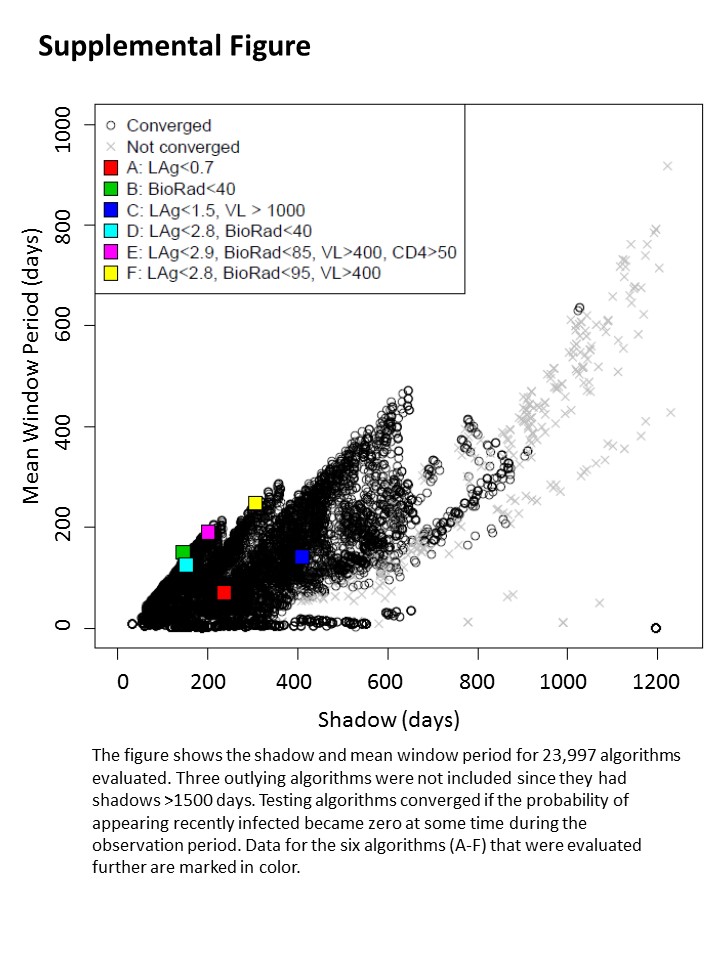

Supplement: Supplementary file 1 — Figure S1. THE figures shows the shadow and mean window period for 23,997 ALgorithms evalated. [file JIA2-21-e25082-s001.jpg]
