# Supplementary material for: Identification and validation of a multi‐assay algorithm for cross‐sectional HIV incidence estimation in populations with subtype C infection
Source: J Int AIDS Soc. 2018 Feb 28;21(2):e25082. doi: 10.1002/jia2.25082 (PMC5829581; doi:10.1002/jia2.25082)
Supplement: Supplementary file 2 — Table S1. The table shows the assay cutoffs and performance characteristics of testing algorithms A through F, and 26 additional testing algorithms. VL: viral load (copies/mL); CD4: CD4 cell count (cells/mm3); values for the window period and shadow are shown in days [file JIA2-21-e25082-s002.docx]

**Supplemental Table**.

| Algorithm | LAg-Avidity | BioRad-Avidity | VL | CD4 | Window Period | Shadow |
| --- | --- | --- | --- | --- | --- | --- |
| A | 0.7 |  |  |  | 71 | 237 |
|  | 1.5 |  |  |  | 222 | 734 |
| B |  | 40 |  |  | 151 | 146 |
|  |  | 80 |  |  | 286 | 519 |
|  |  |  | 1000 |  | 432 | 648 |
|  |  |  |  | 200 | 762 | 1176 |
| D | 2.8 | 40 |  |  | 126 | 152 |
|  | 1.5 | 80 |  |  | 135 | 493 |
|  | 1.5 |  | 400 |  | 156 | 395 |
|  | 2.0 |  | 400 |  | 223 | 449 |
|  | 2.5 |  | 400 |  | 311 | 471 |
| C | 1.5 |  | 1000 |  | 144 | 415 |
|  | 2.0 |  | 1000 |  | 197 | 438 |
|  | 2.5 |  | 1000 |  | 278 | 462 |
|  |  | 40 | 400 |  | 120 | 144 |
|  |  | 80 | 400 |  | 184 | 196 |
|  |  | 40 | 1000 |  | 111 | 131 |
|  |  | 80 | 1000 |  | 165 | 179 |
|  |  |  | 400 | 200 | 449 | 608 |
|  |  |  | 1000 | 200 | 410 | 606 |
|  | 2.0 | 80 | 400 |  | 128 | 166 |
| F | 2.8 | 95 | 400 |  | 248 | 306 |
|  | 2.0 | 80 | 1000 |  | 112 | 137 |
|  | 2.5 | 80 | 1000 |  | 139 | 146 |
|  | 3.0 | 80 | 1000 |  | 165 | 179 |
|  | 1.0 | 95 | 1000 |  | 67 | 193 |
|  | 1.5 | 95 | 1000 |  | 102 | 225 |
|  | 2.0 | 95 | 1000 |  | 141 | 248 |
|  | 2.5 | 95 | 1000 |  | 189 | 260 |
|  | 3.0 | 95 | 1000 |  | 261 | 356 |
| E | 2.9 | 85 | 400 | 50 | 191 | 201 |
|  | 3.0 | 95 | 1000 | 200 | 255 | 353 |

Legend. The table shows the assay cut-offs and performance characteristics of testing algorithms A through F, and 26 additional testing algorithms. VL: viral load (copies/mL); CD4: CD4 cell count (cells/mm^3^); values for the window period and shadow are shown in days.
