# Supplementary material for: Identification and validation of a multi‐assay algorithm for cross‐sectional HIV incidence estimation in populations with subtype C infection
Source: J Int AIDS Soc. 2018 Feb 28;21(2):e25082. doi: 10.1002/jia2.25082 (PMC5829581; doi:10.1002/jia2.25082)
Supplement: Supplementary file 3 — Appendix S1. Supplementary material. [file JIA2-21-e25082-s003.docx]

**SUPPLEMENTARY MATERIAL**

Statistical Methods

This file includes additional details on the statistical methods used to evaluate different testing algorithms. The proportion of samples classified as MAA positive was modeled as a function of calendar time (*t*) since seroconversion, denoted *ϕ(t)*; this value depends on the assays included in the MAA and the cutoff value used for each assay*.* The *mean window period* was calculated as μ=$\int_{0}^{\infty} \phi(t$*)dt* and the *shadow* was calculated as $\int_{0}^{\infty} {}^{-1}t\phi(t$*)dt* (1, 2).

CD4 cell count data were missing for 238 samples of the 2,442 samples; these values were missing completely at random (MCAR); results from these 238 samples were excluded from analyses that included CD4 cell count data (3). Among the remaining 2,204 samples, HIV viral load data were missing for 766 samples. Only 55 of these 766 samples could have been classified as MAA positive (the remaining 711 samples had LAg-Avidity assay results >3 normalized optical density units [OD-n]). The 55 samples with a LAg-Avidity result <3 OD-n were missing viral load data solely because of sample depletion and could therefore be considered conditionally MCAR.

The value for *ϕ(t)* for a specified MAA was estimated by fitting two logistic regressions and multiplying the two curves together. The first regression estimated *Pr(LAg-Avidity <3|Time=t),* using all 2,442 samples (the notation “Pr” means “the probability of”; “|” means “given”). The second regression estimated *Pr(MAA classification = “recent” |Time=t,LAg-Avidity <3)*, using the 733 samples that had complete data for all biomarkers and a LAg-Avidity result <3 OD-n. A cubic spline with a knot at two years was used in the first regression; a cubic polynomial (without a knot) was used in the second regression. A similar method was used in a previous study to handle missing data (4).

References:

1. **Brookmeyer R**. 2010. On the statistical accuracy of biomarker assays for HIV incidence. J Acquir Immune Defic Syndr **54**:406-414.

2. **Konikoff J, Brookmeyer R, Longosz AF, Cousins MM, Celum C, Buchbinder SP, Seage GR, 3rd, Kirk GD, Moore RD, Mehta SH, Margolick JB, Brown J, Mayer KH, Koblin BA, Justman JE, Hodder SL, Quinn TC, Eshleman SH, and Laeyendecker O**. 2013. Performance of a limiting-antigen avidity enzyme immunoassay for cross-sectional estimation of HIV incidence in the United States. PLoS One **8**:e82772.

3. **Little RJA, and Rubin DB.** 2014. Statistical analysis with missing data. Hoboken, NJ: J Wiley & Sons.

4. **Konikoff JM**. 2015. Cross-sectional HIV incidence estimation: Techniques and challenges. Ph.D. Thesis, Univ. of California at Los Angeles.
